# 

**Published:** 1894-09-01

**Authors:** 


### CONTENTS.

**The Costliness of Modern Treatment** - - - - - - 439
**Around the Hospitals** - - - - - - - - - - - 440
**Potassium Ethylate.**—By Sir BENJAMIN WARD RICHARDSON, M.D., F.R.S. - - - - - - - - - - - 441
**The First Century of English Medical Literature.**—VIII. The Earliest Herbals - - - - - - - - - 442
**Notes and News** - - - - - - - - - - - 444
**Medical Progress and Hospital Clinics:**
Fibro-Myomatous Tumours of the Uterus Producing Bladder Symptoms - - - - - - - - - - - 445
The Treatment of Habitual Constipation - - - - - - 446
PROGRESS IN MEDICINE.—Renal Medicine - - - - - - 447
**Annotations.**—The After Care of the Insane—Soap—Roof Gardens for Londoners - - - - - - - - - - - 443
**The British Medical Association at Bristol.**—The Annual Museum - - - - - - - - - - - 449
**New Appliances and Things Medical** - - - - - 450
**The Institutional Workshop:**
The Early History of the Hospital Sunday and Saturday Funds - 451
PRACTICAL DEPARTMENTS.—Cocoas and Chocolates - - - 452
THE STORY OF THE INSANE FROM YEAR TO YEAR - - - 453
CONSTRUCTION NOTES - - - - - - - - - 454
**Scraps and Gleanings** - - - - - - - - 454
**Street Collections.** By HENRY C. BURDETT - - - - - 455

### EXTRA SUPPLEMENT.—THE NURSING MIRROR.

**News from the Nursing World.**—Holidays and News—The Male Nurse—Stewardesses—A Model Time Table—Undesirable Postponements—The Nurse Dolls—District Nurses in Adelaide—Nurses and Patients—Nursing at Guy's—The State Hospitals of England—Occasional Holidays—Short Items - ccxxi

**On General Nursing.**—XXVII. Nursing of Burns - - ccxxiii

**Hospitals in Brazil** - - - - - - - - - - ccxxiii

**Holidays in Japan.** III.—Hot Baths - - - - - - ccxxiv

**Worthing** - - - - - - - - - - - - - ccxxiv

**Where was the Label?** - - - - - - - - - - ccxxiv

**Notes and Queries** - - - - - - - - - - ccxxiv

**The Position of the Workhouse Infirmary Nursing Association** - - - - - - - - - - - ccxxv

**A Case for Assistance** - - - - - - - - - ccxxv

**Minor Appointments—Wants and Workers** - - - ccxxv

**Everybody's Opinion** - - - - - - - - - - ccxxvi

**For Reading to the Sick** - - - - - - - - ccxxvi

**The Book World for Women and Nurses** - - -

